# The Later Stone Age Calvaria from Iwo Eleru, Nigeria: Morphology and Chronology

**DOI:** 10.1371/journal.pone.0024024

**Published:** 2011-09-15

**Authors:** Katerina Harvati, Chris Stringer, Rainer Grün, Maxime Aubert, Philip Allsworth-Jones, Caleb Adebayo Folorunso

**Affiliations:** 1 Paleoanthropology, Senckenberg Center for Human Evolution and Paleoecology, Eberhard Karls Universität Tübingen, Tübingen, Germany; 2 Paleontology Department, Natural History Museum, London, United Kingdom; 3 Research School of Earth Sciences, The Australian National University, Canberra, Australia; 4 Department of Archaeology, University of Sheffield, Sheffield, United Kingdom; 5 Department of Archaeology and Anthropology, University of Ibadan, Ibadan, Nigeria; State University of New York College at Oneonta, United States of America

## Abstract

**Background:**

In recent years the Later Stone Age has been redated to a much deeper time depth than previously thought. At the same time, human remains from this time period are scarce in Africa, and even rarer in West Africa. The Iwo Eleru burial is one of the few human skeletal remains associated with Later Stone Age artifacts in that region with a proposed Pleistocene date. We undertook a morphometric reanalysis of this cranium in order to better assess its affinities. We also conducted Uranium-series dating to re-evaluate its chronology.

**Methodology/Principal Findings:**

A 3-D geometric morphometric analysis of cranial landmarks and semilandmarks was conducted using a large comparative fossil and modern human sample. The measurements were collected in the form of three dimensional coordinates and processed using Generalized Procrustes Analysis. Principal components, canonical variates, Mahalanobis D^2^ and Procrustes distance analyses were performed. The results were further visualized by comparing specimen and mean configurations. Results point to a morphological similarity with late archaic African specimens dating to the Late Pleistocene. A long bone cortical fragment was made available for U-series analysis in order to re-date the specimen. The results (∼11.7–16.3 ka) support a terminal Pleistocene chronology for the Iwo Eleru burial as was also suggested by the original radiocarbon dating results and by stratigraphic evidence.

**Conclusions/Significance:**

Our findings are in accordance with suggestions of deep population substructure in Africa and a complex evolutionary process for the origin of modern humans. They further highlight the dearth of hominin finds from West Africa, and underscore our real lack of knowledge of human evolution in that region.

## Introduction

**Figure 1:**
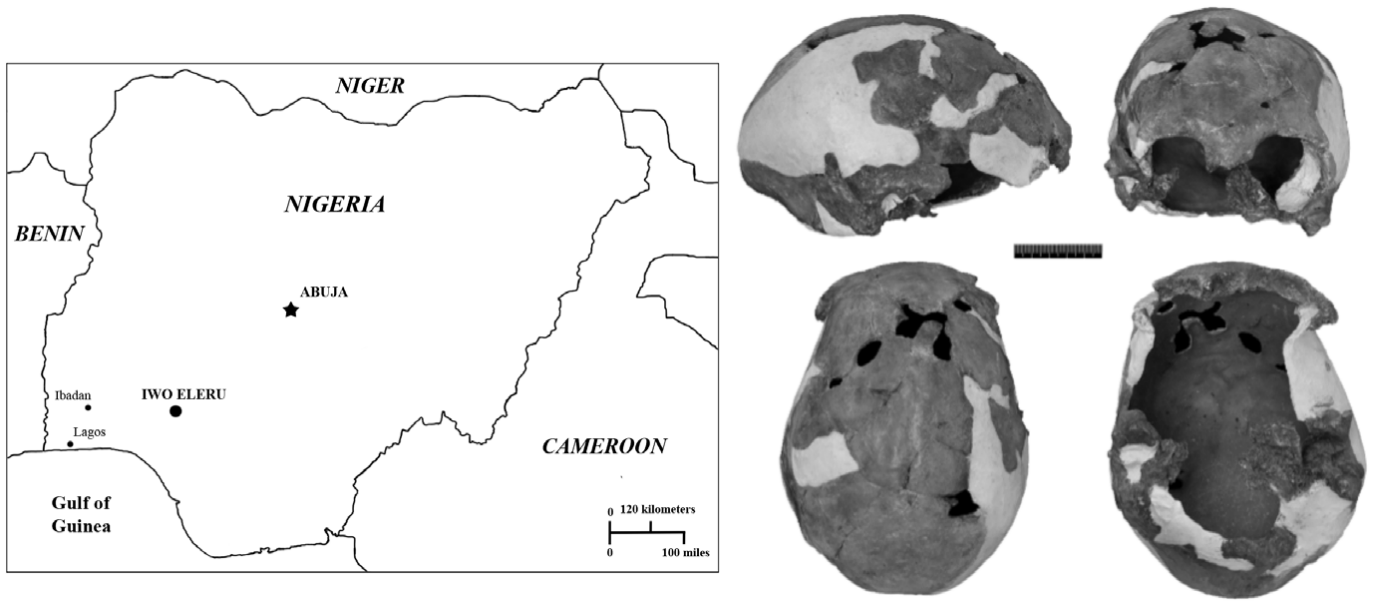
Map of Nigeria, showing the geographic location of the Iwo Eleru rockshelter, and the Iwo Eleru calvaria. Clockwise from top left: Lateral, frontal, ventral and superior views.

The Iwo Eleru burial was excavated from the Iwo Eleru rock shelter, south-western Nigeria, in 1965 by Thurstan Shaw and his team (Figure 1). The skeleton, preserving a calvaria, mandible and some postcranial remains, was found at a depth between 82 and 100 cm from the surface in an undisturbed Later Stone Age (hereafter LSA) context. Radiocarbon analysis of charcoal from the immediate vicinity of the burial resulted in an age estimate of 11,200±200 BP (∼13 ka calibrated). The skull was reconstructed and studied by Brothwell [1] (Figure 1)], who linked it to recent West African populations, though he recognized that its lower vault and frontal profile were unusual, and that the mandible was robust. The specimen is complete along the entire midline from nasion to beyond opisthocranion. Although it slightly asymmetric it shows no major distortions and the relatively well preserved mandible constrains its basal breadth. A preliminary multivariate analysis of cranial measurements by Peter Andrews (in [1]) suggested that the Iwo Eleru specimen was distinct from recent African groups.

**Figure 2:**
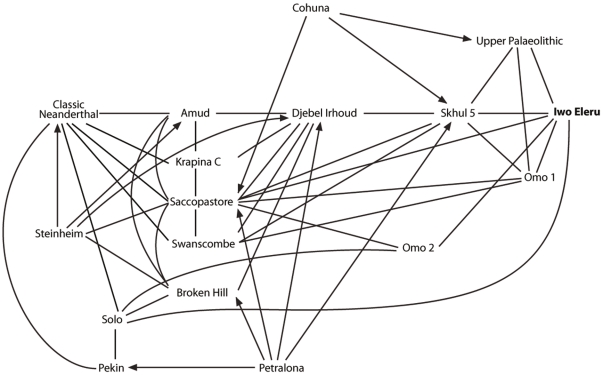
Visualization of the results of Stringer's multivariate analyses [**2]**, [3****], showing the position of the Iwo Eleru calvaria. Mutually close specimens are joined by lines, but an arrowed line indicates where the proximity is not mutual. For example Saccopastore is a nearest neighbour to Petralona, but Petralona is not a near neighbour of Saccopastore. Redrawn with permission from [3].

A more extensive analysis of the cranial measurements of the original Iwo Eleru specimen was conducted by Chris Stringer, who included this cranium in univariate and multivariate (Canonical Variates, Generalised Distance) analyses for his doctoral thesis [2], [3]. Coefficients of separate determination in a cranial analysis using 17 of Howells' measures showed that the main discriminators from an Upper Paleolithic sample were low frontal subtense, low vertex radius, high cranial breadth, high bifrontal breadth, high cranial length and low parietal subtense, against Neanderthals they were primarily low supraorbital projection, low frontal fraction, high parietal chord, high frontal chord, low frontal subtense and low vertex radius, while against Zhoukoudian *Homo erectus* they were low supraorbital projection, high parietal chord, high bifrontal breadth, high vertex radius, high frontal chord and low frontal subtense. Overall it appeared that the cranium was “modern” in its low supraorbital projection, and long frontal and parietal chords, but “archaic” in its high cranial length, low vertex radius, and low frontal and parietal subtenses. Stringer's results highlighted apparent archaic aspects in the specimen in its long and rather low cranial shape, and although modern overall, it also resembled fossils such as Omo Kibish 2, Saccopastore 1 and Ngandong in several respects, falling closer to them than to Upper Palaeolithic and recent samples in some analyses (Figure 2).

In light of the redating of the LSA to a much deeper time depth than originally thought, and of the scarcity of LSA human skeletal remains from Africa in general and from West Africa in particular, we undertook a renewed study of the Iwo Eleru cranium with the aim of better determining its affinities and geological age [4], [5]. A primary replica of the cranial vault of the Iwo Eleru specimen, produced before its return to Nigeria, was digitized by one of the authors (KH). Comparisons of Stringer's measurements on the original and the replica show a maximum discrepancy of 1 mm, suggesting the replica accurately reflects the original shape of the cranium. The 3-D coordinates collected were included in an extensive comparative dataset of Middle, Late Pleistocene and Holocene humans, and a multivariate statistical analysis was undertaken with the goal of assessing its affinities and phylogenetic / population relationships in the context of geographic and temporal human cranial variation. Furthermore, in order to check the possibility that the associated radiocarbon age did not date the specimen, one of us (AF) provided a long bone cortical fragment approximate 1 cm square for a new age estimate. Unfortunately the lack of collagen prevented a direct radiocarbon determination at the Oxford Radiocarbon Accelerator, so Uranium-Series dating of the fragment was carried out instead.

## Results

### Morphometric analysis

**Figure 3:**
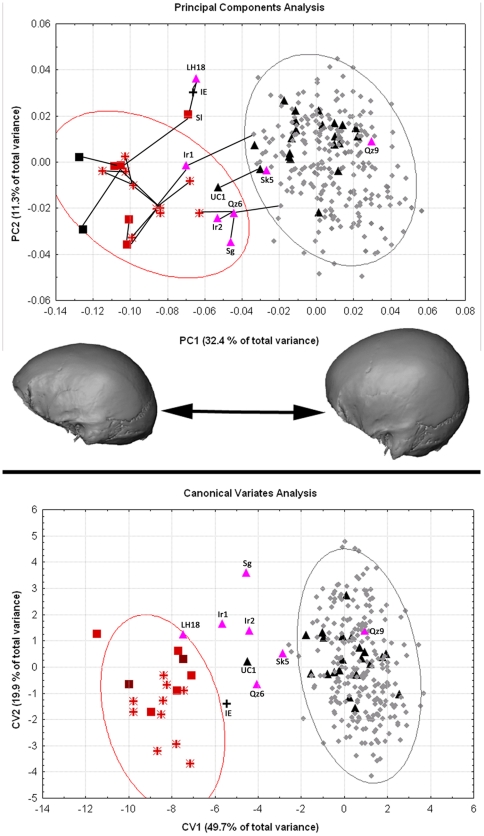
Results of the multivariate statistical analysis of landmarks and semilandmarks. Top: Principal components analysis, PC1 and 2. Cranial shape differences along PC 1 are shown below the graph. The top graph shows a Minimum Spanning Tree of the Inter-individual Procrustes distances for the fossil specimens (black lines connecting specimens). Specimen labels as in Table 1. Bottom: Canonical variates analysis, CV 1 and 2. Symbols: Grey diamonds. Modern humans; Black up triangles: Upper Paleolithic modern humans; Purple up triangles: Late Pleistocene African and Near Eastern hominins; Red stars: *H. neanderthalensis*; Red squares: *H. heidelbergensis* (*s.l*.); Black squares: *H. erectus* (*s.l*.). Ellipses indicate 95% confidence ellipses for Neanderthals (red) and modern humans (gray).

The results of the principal components analysis (PCA) were similar to those described previously for similar neurocranial datasets analyzed by one of us (KH; Figure 3; [6]–[8]). The first principal component accounted for 32.4% of the total variance and separated archaic from modern human specimens. The two *H. erectus* (*s.l*.) specimens fell at the extreme negative of this axis, followed by Neanderthals and *H. heidelbergensis* (*s.l*.). Modern human populations were characterized by more positive scores on PC 1, and there was only minimal overlap among their 95% confidence ellipses and that of the Neanderthals. The Middle-Late Pleistocene African specimens (LH 18, Singa, Djebel Irhoud 1 and 2) and the early modern human specimens from Qafzeh and Skhul fell in the intermediate zone between Neanderthals / *H. heidelbergensis* on one hand and modern humans on the other. Qafzeh 9 was the exception, falling on the positive end of PC 1 and close to Upper Paleolithic European specimens. The latter sample, which included some of the earliest modern human specimens in Europe (Mladeč 1 and 5, Oase 2, Muierii 1, Cioclovina), clustered within the modern human range of variation, and not in the zone of overlap with the archaic specimens. One of the two specimens from Zhoukoudian Upper Cave (UC 101) had a more negative PC 1 score similar to that of Qafzeh 6 and Jebel Irhoud 2 (see also [8]). Iwo Eleru showed a similarly negative PC 1 score, falling closest to LH 18, Saldanha (Elandsfontein) and Spy 2 along this axis. PC 1 reflected differences in the shape of the neurocranium from an elongated, low vault and large, evenly thick, supraorbital torus to a rounded, antero-posteriorly shorter vault, and thinner supraorbital torus with differentiated medial and lateral segments.

**Table 1:** Fossil comparative samples used in the analysis.

**European – W. Asian H. neanderthalensis (n = 10; NEA)**
Amud 1 (Am1), Feldhofer 1* (Fh1), Guattari (Gt) La Chapelle-aux-Saints (Ch), La Ferrassie 1 (Fr1), La Quina 5 (Qn), Shanidar 1* (Sh1), Spy 1& 2 (Sp1, Sp2), Tabun 1 (Tb1)
**Homo heidelbergensis s.l. (n = 5; HH)**
Dali* (Da), Kabwe (Broken Hill) (Kb), Petralona (Pe), Saldanha (Elandsfontein) (Sl), Sima de los Huesos 5* (Sm5)
**Homo erectus s.l. (n = 2; HE)**
KNM-ER 3733 (ER3733), KNM-ER 3883 (ER3883)
**Late Middle-Late Pleistocene fossils from Africa and the Levant (n = 7; LPA, EAM)**
Irhoud 1& 2 (Ir1, Ir2), Ngaloba (LH18), Qafzeh 6* & 9 (Qz6, Qz9), Singa (S1), Skhul 5 (Sk5)
**Upper Paleolithic Eurasian modern humans (n = 20; EUP), Zhoukoudian Upper Cave (n = 2; UC)**
Abri Pataud (AP), Brno 1 (Bn1), Chancelade (Cn), Cioclovina (Ci), Cro Magnon 1, 2, 3 (CM1, CM2, CM3), Dolní Věstonice 3, 13, 15, 16 (Dv3, Dv13, Dv15, Dv16), Grimaldi 4* (Gr), Mladeč 1, 5 (Ml1, Ml5), Muierii (Mu), Oase 2 (Oa), Ohalo II* (Oh2), Pavlov (Pv), Předmostí 3*, 4* (Pd3, Pd4), Upper Cave 101* &103 * (UC1, UC3)

*Indicates specimens for which high-quality casts or stereolithographs were measured. The symbols for each specimen used in the Figures are indicated in parentheses. One of the authors (Stringer) regards Sima de los Huesos 5 as an early Neanderthal rather than a *H. heidelbergensis*.

PC 2 (11.3% of the total variance, Figure 3) appeared to reflect variation among modern humans, with the sub-Saharan African, the Khoisan, Oceanic and Upper Paleolithic samples clustering around zero, and on the positive end of this axis. The Andaman sample was restricted to the negative side, while the Inuit fell around zero. The Europeans, N. Easterners, Asians and Iberomaurusians spanned the entire length of PC 2. The LPA sample was also quite spread out along this axis, while the Neanderthals, *H. erectus* and *H. heidelbergensis s.l*. were relatively centered around zero and on the negative side. Iwo Eleru was once more placed closest to LH 18 and Saldanha. Crania with relatively negative scores on PC 2 displayed antero-posteriorly short and medio-laterally wide shapes, while those with positive scores showed antero-posteriorly long and medio-laterally narrow vaults.

Iwo Eleru generally showed large distances to the other specimens in this analysis. It displayed the shortest inter-individual Procrustes distance to LH18 (0.080), and next closest to La Chapelle-aux-Saints (0.087) and a recent Australian (0.089). LH18 itself was closest to the Middle Pleistocene Saldanha individual (0.059). It also showed relatively small Procrustes distances to two Upper Paleolithic Europeans, Oase 2 (0.071) and Predmostí 3 (0.074). La Chapelle showed the shortest distances to other Neanderthals: Guattari (0.054), La Quina 5 (0.054), Spy 1 (0.057), Amud 1 (0.061), La Ferrassie 1 (0.067), Feldhofer 1 (0.075), Shanidar 1 (0.076); as well as to older specimens: Sima de los Huesos Cranium 5 (0.069), Dali (0.060), Irhoud 1 (0.067). The Procrustes inter-individual distances were used to generate a minimum spanning tree for the fossil specimens on the plot of PC 1 and 2 (Figure 3).

The results of the canonical variates analysis (CVA) were consistent with those of the PCA (Figure 3). The first canonical axis (49.7%) separated archaic from modern specimens, with late archaic or early modern humans (the Irhoud specimens, Qafzeh 6, Singa, LH18) generally falling in an intermediate position. Iwo Eleru, as well as Upper Cave 101, also fell in this region, with the former and LH18 being just at the outskirts of the Neanderthal confidence ellipse. The Mahalanobis squared distances among the predefined groups are reported in Table S2. Iwo Eleru showed large distances from all other groups. The smallest distance was to the Upper Cave specimens, themselves a very small group of just two individuals. Relatively small squared distances were also shown between Iwo Eleru and the Qafzeh-Skull group, Neanderthals and *H. erectus* (*s.l*.).

**Figure 4:**
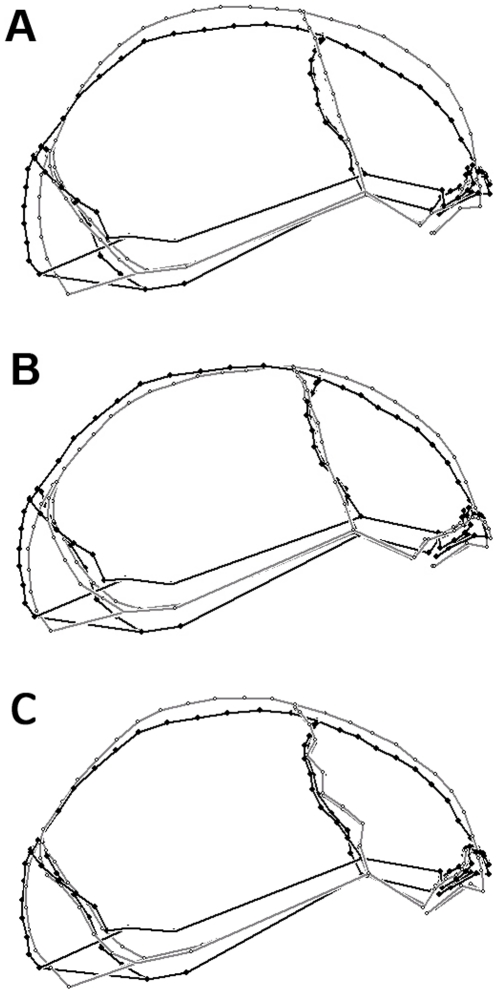
Shape comparisons of Iwo Eleru. Iwo Eleru (black) compared to the modern human mean configuration (A, gray), to the Late-Middle Pleistocene African mean configuration (B, gray), and to its closest modern human neighbor in overall shape (C, gray).

The departure of Iwo Eleru from the modern human average cranial shape was further underlined by the comparison of its landmark configuration to the mean configuration of modern humans (Figure 4A). Iwo Eleru was characterized by a more elongated cranial vault and flattened frontal and parietal bones. Its browridge was also slightly more forward projecting than the average modern human shape. Iwo Eleru was more comparable to the mean LPA landmark configuration in its elongated and low cranial shape and the degree of browridge projection (Figure 4B). Its nearest recent human neighbor, an Australian female (Figure 4C), also showed a relatively low vault and pronounced browridges. However, the latter specimen exhibited an overall more curved sagittal profile than Iwo Eleru, with a more steeply rising frontal bone, an expanded and more curved parietal and a more rounded occipital with a lower position of inion, all typical modern human conditions.

### Uranium-Series Dating

The elemental U and Th values vary greatly along the profiles of the bone sample from the Iwo Eleru skeleton (left-hand diagrams in Figures S1 and S2). The outer surfaces and pores show measureable Th concentrations, which indicate the presence of detrital material. There is a gradient of higher U-concentrations in the centre of the bone (around 80 to 100 ppm) towards the outsides where U-concentrations of 50 to 60 ppm were measured. These lower U-concentrations may either be due to a denser bone structure, with less internal surface area for adsorbing uranium, or to leaching of U from the volumes close to the surface. The apparent U-series age estimates vary between about 10 to 14 ka in the central part of the bone and increase in some laser scans to between about 15 to 17 ka closer to the surface. The right-hand columns of Figures S1 and S2 show the plot of apparent U-series age versus U-concentration.

It is expected from the diffusion-adsorption model for U-uptake [9]–[12] that spatially resolved analyses across a homogeneous bone yield u-shaped or constant U-concentration and apparent U-series age profiles. In ideal circumstances, a plot of apparent U-series age versus U-concentration would either be flat or show increasing U-series age estimates with increasing U-concentrations. This is clearly not the case for the results on Iwo Eleru (Figures S1 and S2, right hand columns). The apparent U-series age estimates are more or less constant in the inner part of the bone fragment and increase towards the outside while the U-concentrations decrease.

One of the problems of U-series dating of bone is that the different domains within a bone may give very different U-series ages. The best age estimate is actually not derived by simply averaging all results, but from identifying the domains that had experienced the fastest U-uptake and have remained a closed system since. This may only apply to very small volumes (see [13]). The U-series analyses of the central part of the bone give a minimum age of the sample. Excluding any results with more than 10 ppb Th (indication of contamination) and U concentrations of less than 75 ppm, all scans give compatible results with mean ages ranging between 10.6±1.7 (Track 2) and 12.7±1.6 ka (Track 4), see right hand columns in Figures S1 and S2. Most apparent U-series age results from the central parts of the bone are within 9 and 14 ka. Virtually all age results overlap within their errors. Most of the scatter is likely due to statistical variation. The best age estimate is thus derived from the mean value of all Th-free age estimates in the central part of the bone (11.7±1.7 ka). This age estimate is derived from more than 80% of the exposed bone area (excluding the pores), which may be taken as an indication that most of the uranium was accumulated within a relatively short time range. The average U-series age increases by less than 1 ka (i.e. less than 10%) by including the Th-free results of the domains with lower U-concentrations. Considering that the outer U-concentrations are lower by up to a factor of four compared to the interior part, U-leaching is apparently not the main cause for the reduced U-concentrations.

The question is whether the specimen could be substantially older. Most older apparent age estimates are associated with relatively high Th and low U concentrations, either close to the outside or in pores (Tracks 1, 3, 4, 11, and 12). Here, the older apparent U-series results may well have been influenced by detrital material as well as U-leaching. Other older results (>15 ka) are seen for example in Track 8 (around cycle 3100), Track 11 (around cycle 430 and 550) and Track 12 (around cycle 1480), see arrows in the respective diagrams in Figure S2. In Track 12, the section between cycle 1481 and 1488 yields an age of 19.3±1.8 ka. The immediately adjacent sections yield 13.9±2.6 ka (cycles 1472 to 1481) and 10.8±2.1 ka (cycles 1488 to 1505). It is a simple statistical fact that some subsamples of a single population will deviate from the other results by more than 2-σ. The most likely apparent age result comes from the average of this section. The same seems to apply to the other marked sections.

Nevertheless, Tracks 9 (between cycles 3076 and 3161) and 11 (between cycles 726 and 791, see circles in the respective diagrams of Figure S2) show wider domains with relatively old apparent U-series ages (15.0±1.3 and 16.3±0.5 ka), which do not depend on the U-variations within these domains. While U-leaching cannot entirely be excluded in these sections, the lower U-concentrations may just as well be due to different amounts of internal surface area. These two sections are close to the lower surface and could have preserved the original U-series isotope signatures. If this is the case, the most likely age of the bone is around 16.3 ka. In summary, the minimum age of the bone is around 11.7±1.7 ka while some domains indicate an age as old as 16.3±0.5 ka.

## Discussion

Our analysis indicates that Iwo Eleru possesses neurocranial morphology intermediate in shape between archaic hominins (Neanderthals and *Homo erectus*) and modern humans. This morphology is outside the range of modern human variability in the PCA and CVA analyses, and is most similar to that shown by LPA individuals from Africa and the early anatomically modern specimens from Skhul and Qafzeh. Iwo Eleru is distinct from the recent African samples used here (although the range of recent modern human variation encompasses relatively low and elongated cranial shapes approaching this condition). Past work has suggested that neurocranial shape reflects population history relatively reliably among modern human populations [14], [15]. Although we did not find unambiguous strong affinities between Iwo Eleru and the samples used here, its overall morphological similarities with early modern humans suggest a link to these early populations and possibly a late Middle-early Late Pleistocene chronology. Nonetheless, the archaeological setting, stratigraphy, previous radiocarbon [see 4] and our new U-series dating indicate a much younger, terminal Pleistocene age for this cranium. Such a late chronology for the Iwo Eleru cranium implies that the transition to anatomical modernity in Africa was more complicated than previously thought, with late survival of “archaic” features and possibly deep population substructure in Africa during this time.

Thus our restudy of the Iwo Eleru cranium confirms previously noted archaic cranial shape aspects, and the U-series age estimates on its skeleton support the previously proposed terminal Pleistocene date for this burial. Our findings also support suggestions of deep population substructure in Africa and a complex evolutionary process for the origin of modern humans [16], [17], [7], [18], [19], [20], [21]. Perhaps most importantly, our analysis highlights the dearth of hominin finds from West Africa, and underscores our real lack of knowledge of human evolution in that region, as well as others. As also indicated by restudy of the Ishango (Congo) fossils [22], Later Stone Age fossils from at least two regions of Africa retain significant archaic aspects in their skeletons. We hope that the next stage of this research will extend studies to the Iwo Eleru mandible and postcrania, and to comparative materials such as those from Ishango.

## Materials and Methods

### Morphometric analysis

The comparative sample for this analysis comprises several Pleistocene human fossils from Africa and Eurasia, and two hundred and forty two recent human crania representing nine broad geographic groups (Tables 1 and 2). The sex of the modern human crania was assigned on the basis of museum catalogue records, cranial morphology and size and, in the rare cases of associated postcrania, pelvic morphology. In as much as possible, male and female samples were balanced for sample sizes. Since such sex assignment is imperfect for recent humans, and even more problematic for fossil specimens, sexes were pooled in the analyses. Original fossils were measured, with the exception of few cases where the originals no longer exist or were unavailable for study. In those few instances, high quality casts or stereolithographs from the collections of the Division of Anthropology of the American Museum of Natural History, the Department of Anthropology at New York University, the Institut de Paléontologie Humaine, and the Department of Human Evolution at the Max Planck Institute for Evolutionary Anthropology, were measured. Using casts as alternatives to fossil specimens is an imperfect solution but one that is necessary in cases where the originals are not available for study, or have been destroyed.

**Table 2:** Recent human comparative samples.

Recent human samples	
Sub-Saharan African (AFR; Kenya, Zulu; sub-recent; NHM, WITS)	*n* = 27
Andamanese (AND; Andaman Islands; sub-recent; NHM)	*n* = 28
Asian (AS; China, Thailand; sub-recent; MH)	*n* = 39
Oceanic (OCE; Australia; sub-recent; NHM)	*n* = 26
Khoisan (KHO; South Africa; Holocene; IZCT, UCT)	*n* = 58
Inuit (IN; Alaska, Greenland; sub-recent; AMNH)	*n* = 14
Europe (EUR; sub-recent; IAL)	*n* = 15
Near East (NE; Syria; sub-recent; MH)	*n* = 20
Iberomaurusian (IB; Morocco; Holocene; IPH)	*n* = 15
**Total**	**n = 242**

Museum abbreviations: AMNH: American Museum of Natural History, New York; IAL: Institute of Anatomy, Leipzig; IPH: Institut de Paléontologie Humaine, Paris; IZCT: Iziko Museums of Cape Town; MH: Musée de l′Homme, Paris; NHM: Natural History Museum, London; UCT: University of Cape Town; WITS: University of the Witwatersrand, Johannesburg.

The population labels used in the Figures, the geographic and temporal provenience of the samples, and the museums where these samples are housed are indicated in parenthesis.

Data were collected in the form of three-dimensional coordinates of neurocranial osteometric landmarks, defined as homologous points that can be reliably and repeatedly located, using a Microscribe [23] portable digitizer (Table S1). Landmarks along the midsagittal profile from glabella to inion, along the coronal and lambdoid sutures, and along the upper margin of the supraorbital torus were also registered (Table S1). The points along these outlines were automatically resampled to yield the same semilandmark count on every specimen [24], [6]. These points were chosen so as to reflect the neurocranial morphology of the fossils as fully as possible.

The data were processed using geometric morphometric methods (GMM), which preserve the geometry of the object studied better than traditional measurements, and thus allow for a better analysis of shape. These techniques also readily account for size correction and enable visualization of the shape changes between specimens in specimen space. Perhaps most importantly, they allow the quantification of some anatomical features that are difficult to measure conventionally. Because of these qualities, GMM have gained widespread and increasing use in the recent literature on human variation. Despite these general advantages of GMM, they do not accommodate missing data, often necessitating some level of data reconstruction in fossil studies. Therefore landmarks on specimens with minimal damage were estimated during data collection, using anatomical clues from the preserved surrounding areas. Bilateral landmarks and curves missing on one side were further reconstructed by superimposing the landmark configurations of specimens with missing data with their reflections, and by substituting the coordinates for each missing landmark with the fitted homologous counterpart on the other side. This is a process known as ‘reflected relabeling’ [25]. Further data reconstruction was allowed in the case of LH 18, an important specimen with only minimal damage (frontomalare temporale is missing on both sides). Semilandmarks were ‘slid’ in Mathematica [26] using routines developed by Philipp Gunz and Philipp Mitteroecker [27]; for additional details see also [28], [6].

Landmarks and slid semilandmarks were superimposed with Generalized Procrustes Analysis (GPA) using the Morpheus software package [29]. The fitted coordinates were then analyzed statistically using principal components analysis (PCA), canonical variates analysis, Procrustes distances, and Mahalanobis squared distances. These statistics were calculated with the software packages SAS [30], NTSys [31], and TPSsmall (version 1.20; [32]). Visualization of shape differences along principal components axes was achieved with the use of the EVAN toolbox (EVAN society). The pattern of variation in the sample was evaluated through the PCA, and the similarities among specimens were assessed using inter-individual Procrustes distances (defined as the square root of the sum of squared distances between two superimposed landmark configurations). Similarities and differences among groups were evaluated using the CVA, Mahalanobis D^2^ and mean Procrustes distances between groups. For the purposes of these analyses the fossil samples were partitioned in the following groups: *H. neanderthalensis* (NEA); *H. heidelbergensis sensu lato*; Late Pleistocene Africans (LPA); Early anatomically modern humans from Qafzeh and Skhul (EAM); Upper Paleolithic Europeans (EUP); and Zhoukoudian Upper Cave 101 and 103 (UC). The Mahalanobis statistic represents the morphological difference among groups, scaled by the inverse of the pooled within-group covariance matrix. The larger the values of the D^2^ distance, the farther the group centroids are from each other. Mahalanobis D^2^ assumes equality of covariance, and therefore might be affected by violations of this assumption (though see [33] for a discussion of this issue). Procrustes distance, on the other hand, does not account for non-independence of landmark coordinates and within-group variation. It reflects differences in total shape, and does not take into account patterns of covariation. It is therefore not affected by the assumption of equality of covariance. The first 15 principal components (all components accounting for >0.1% of the total variance; taken together they account for 82.4% of the variance) were used as variables in the CVA and Mahalanobis analyses in order to reduce the number of variables. Because sample sizes were not equal, a correction in calculating this statistic was used, following [34]. Finally, morphological similarity was assessed visually by comparing the landmark configuration of Iwo Eleru, after size correction, to the mean landmark configurations of other groups, using the software package Morpheus [25].

### Uranium-series analysis

In order to obtain an age estimate on the human remains from Iwo Eleru, a postcranial bone was directly analysed for dating. A diamond wire saw was used to cut a plane cross section about 2 mm away from the surface. The central part of the bone shows large pores which are filled with detritus. The upper 2 mm contain many smaller pores while the lower 1.5 to 2 mm consist of dense bone with few, small pores. Twelve laser ablation scans were then recorded on the cross-section (Figure S3), with the scan direction perpendicular to the outer surfaces. Laser ablation U and U-series analyses were carried out at Australian National University; for the experimental set-up see [35], [36] and for applications on human fossils [37], [38], [13].

## Supporting Information

Figure S1 Summary of elemental and U-series analysis for Tracks 1 to 6. Left hand panels: U, and Th elemental concentrations and age calculations. Right hand panels: Relationship between calculated age and U-concentration.
(TIF)

Figure S2 Summary of elemental and U-series analysis for Tracks 7 to 12. Left hand panels: U, and Th elemental concentrations and age calculations. The age results indicated by arrows and circles are discussed in the text. Right hand panels: Relationship between calculated age and U-concentration.
(TIF)

Figure S3 Cross section of the bone sample used for laser ablation U and Th elemental as well as U-series analysis. Arrows indicate the position of the scans shown in Figures S1 and S2.
(TIF)

Table S1 Landmarks and semi landmarks used in the analysis
(DOCX)

Table S2 Mahalanobis D^2^ among groups used in this study. Below the diagonal are values corrected for unequal sample sizes. Sample labels as in Tables 1 and 2.
(DOCX)
