# Correction: The Later Stone Age Calvaria from Iwo Eleru, Nigeria: Morphology and Chronology

**DOI:** 10.1371/annotation/887b6c18-6c37-44d2-8a50-2760bc9ad5d6

**Published:** 2013-11-07

**Authors:** Katerina Harvati, Chris Stringer, Rainer Grün, Maxime Aubert, Philip Allsworth-Jones, Caleb Adebayo Folorunso

During a recent examination of our morphological 3-D coordinate dataset, it was ascertained that it included a subadult specimen not listed in the publication. This specimen was not shown in the plots but mistakenly remained in the analysis as part of the early modern human sample (EAM). We thank Dr. Waddell for bringing this issue to our attention.

We have re-analyzed our data excluding this specimen. Although some of the numerical results change slightly, the main findings, relationships among samples and our conclusions do not change. The PCA and CVA plots, as well as the minimum spanning tree, are nearly identical to those originally reported (Corrected Figure 3). The new Mahalanobis D2 results are similar in the values obtained and pattern of relationships among samples to the ones originally reported (Corrected SI Table 2). The main difference is the distance between Iwo Eleru and the early modern human Qafzeh-Skhul sample from the Levant (EAM), which dates to between 90-130 ka, in the distances corrected for unequal sample sizes. This group, and not the much later Zhoukoudian Upper Cave sample as originally reported, is the closest neighbor to Iwo Eleru in the corrected Mahalanobis D2. This result, although different from that originally reported, strengthens our conclusion that Iwo Eleru bears greater morphological similarity to early rather than late modern human samples from Africa and Eurasia.

The revised dataset and the updated key for the labels used in the dataset are also provided with this Correction.

Figure 3: 

**Figure:**
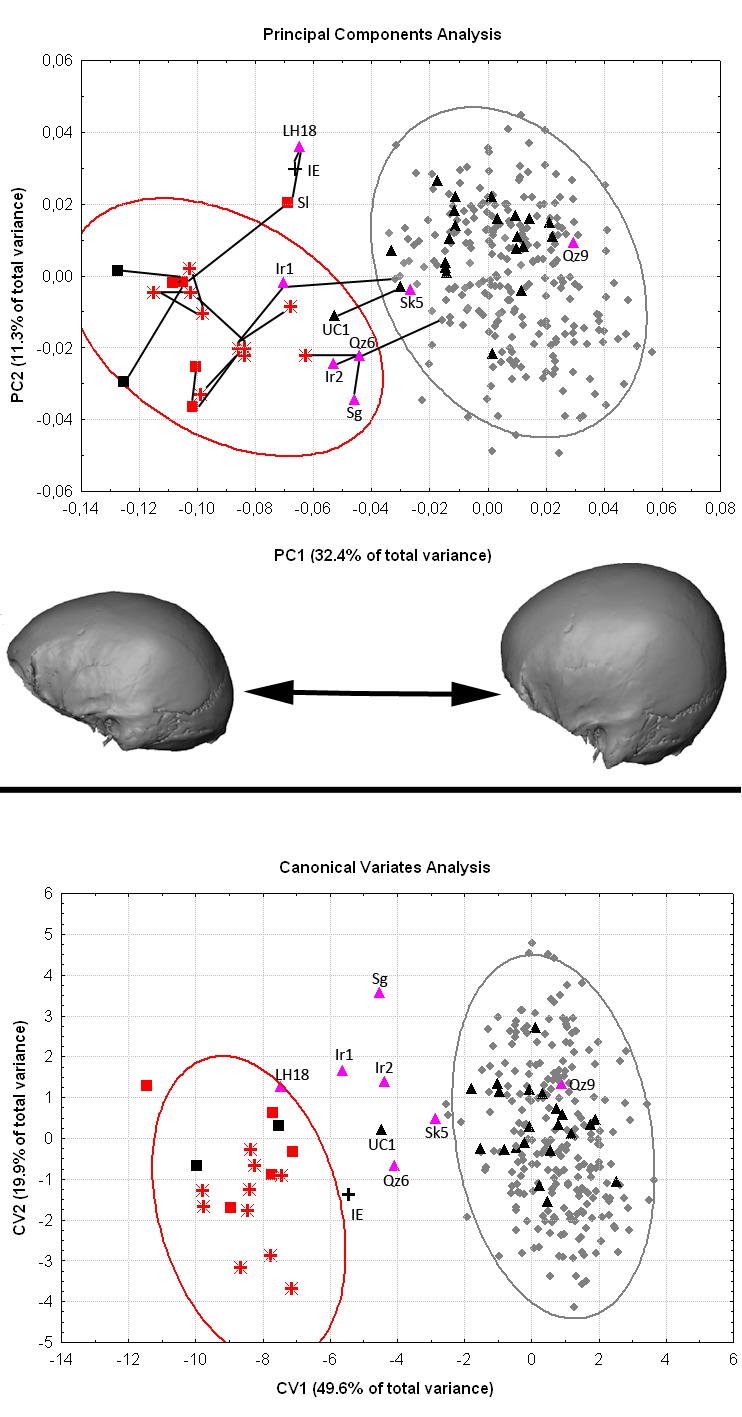


SI Table 2: 

Dataset: http://www.plosone.org/corrections/pone.0024024.001.cn.txt

Label Key: http://www.plosone.org/corrections/pone.0024024.002.cn.doc

