# Supplementary figures and images for: The Later Stone Age Calvaria from Iwo Eleru, Nigeria: Morphology and Chronology
Source: PLoS One. 2011 Sep 15;6(9):e24024. doi: 10.1371/journal.pone.0024024 (PMC3174138; doi:10.1371/journal.pone.0024024)

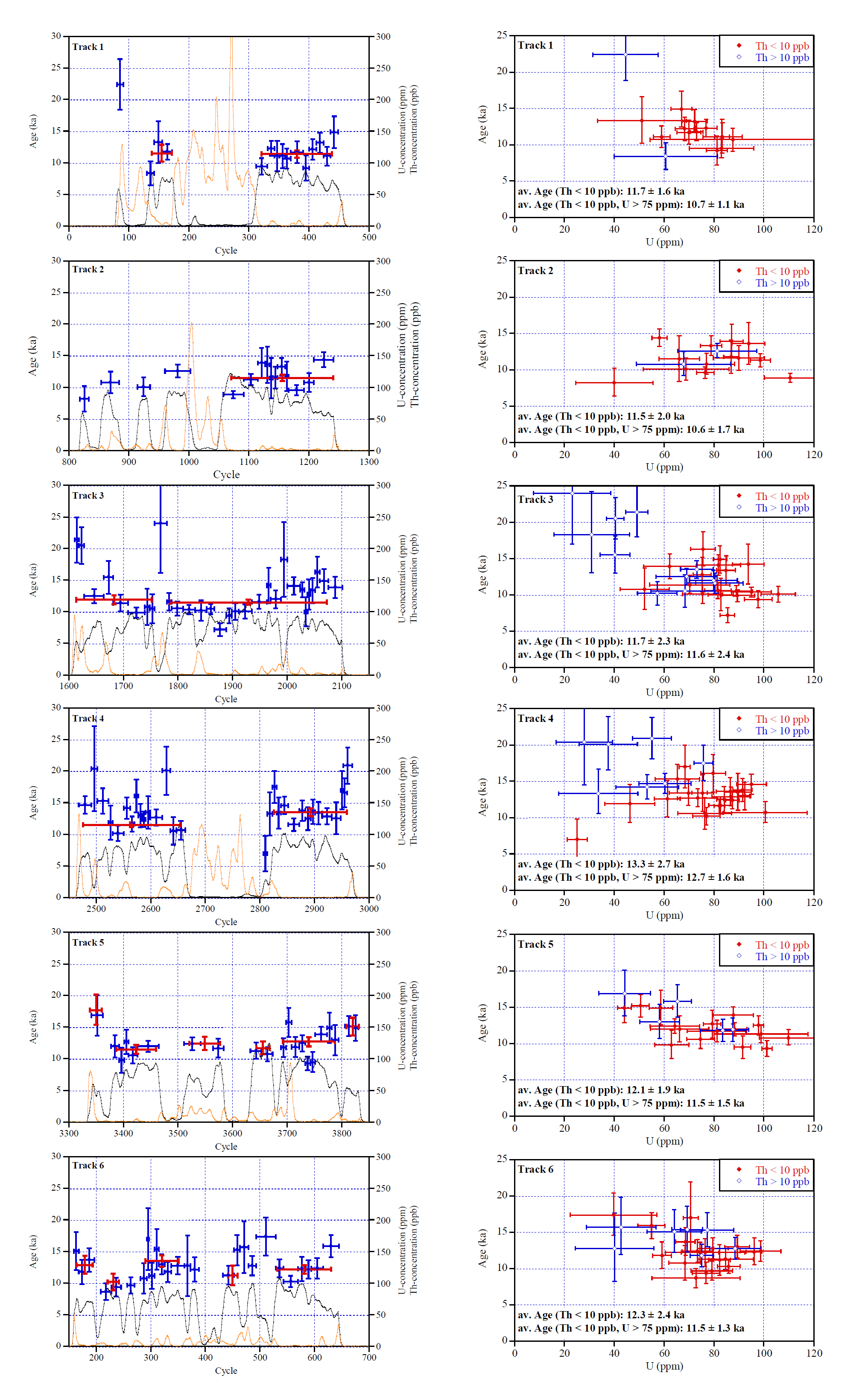

Supplement: Figure S1 — Summary of elemental and U-series analysis for Tracks 1 to 6. Left hand panels: U, and Th elemental concentrations and age calculations. Right hand panels: Relationship between calculated age and U-concentration. (TIF) [file pone.0024024.s001.tif]

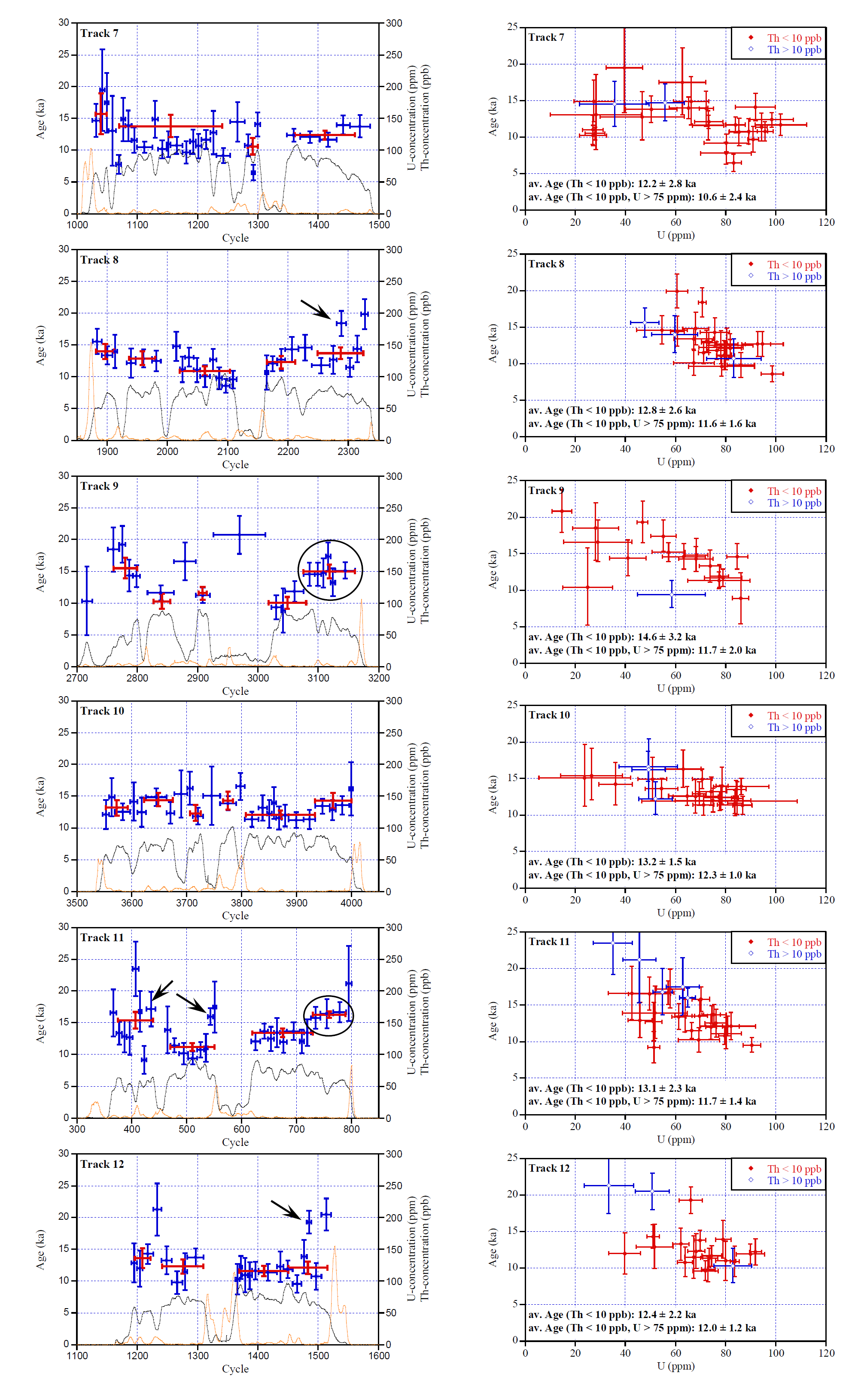

Supplement: Figure S2 — Summary of elemental and U-series analysis for Tracks 7 to 12. Left hand panels: U, and Th elemental concentrations and age calculations. The age results indicated by arrows and circles are discussed in the text. Right hand panels: Relationship between calculated age and U-concentration. (TIF) [file pone.0024024.s002.tif]

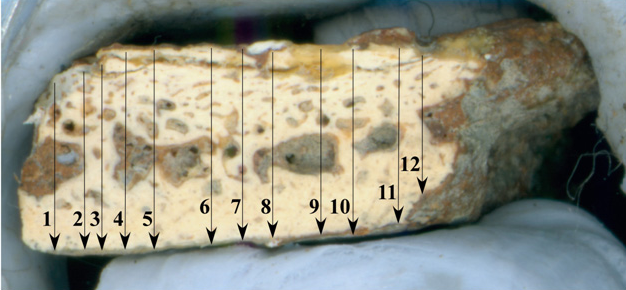

Supplement: Figure S3 — Cross section of the bone sample used for laser ablation U and Th elemental as well as U-series analysis. Arrows indicate the position of the scans shown in Figures S1 and S2. (TIF) [file pone.0024024.s003.tif]
