# Supplementary material for: The Later Stone Age Calvaria from Iwo Eleru, Nigeria: Morphology and Chronology
Source: PLoS One. 2011 Sep 15;6(9):e24024. doi: 10.1371/journal.pone.0024024 (PMC3174138; doi:10.1371/journal.pone.0024024)
Supplement: Table S1 — Landmarks and semi landmarks used in the analysis (DOCX) [file pone.0024024.s004.docx]

**Table S1: Landmarks and semi landmarks used in the analysis**

| **LANDMARKS** |
| --- |
| 1. Glabella (GLA); 2. Post-toral Sulcus (Minima of concavity on midline of post-toral frontal squama); 3. Bregma; 4. Lambda (LBD); 5. Inion (IN); 6, 7. Mid-orbit Torus Superior (Right and Left; points on superior aspect of supraorbital torus, approximately at the middle of the orbit);8, 9. Mid-orbit Torus Inferior (Right and Left; points on inferior margin of supraorbital torus, approx. at the middle of the orbit); 10, 11. Frontomalare Orbitale (FMO; Right and Left); 12, 13. Frontomalare Temporale (FMT; Right and Left); 14, 15. Anterior Pterion (PTE; Right and Left); 16, 17. Parietal Notch (Right and Left); 18, 19. Asterion (AST; Right and Left). |
| **RIDGE CURVES** |
| 1. Supraorbital torus (21 semilandmarks from FMT Right to FMT Left); 2. Midsagittal profile (26 semilandmarks from GLA to IN); 3. Coronal suture (20 semilandmarks from PTE Right to PTE Left); 4. Lambdoid suture (14 semilandmarks from AST right to LBD to AST Left). |
