# Supplementary material for: The Later Stone Age Calvaria from Iwo Eleru, Nigeria: Morphology and Chronology
Source: PLoS One. 2011 Sep 15;6(9):e24024. doi: 10.1371/journal.pone.0024024 (PMC3174138; doi:10.1371/journal.pone.0024024)
Supplement: Table S2 — Mahalanobis D2 among groups used in this study. Below the diagonal are values corrected for unequal sample sizes. Sample labels as in Tables 1 and 2. (DOCX) [file pone.0024024.s005.docx]

**Table S2: Mahalanobis D^2^ among groups used in this study. Below the diagonal are values corrected for unequal sample sizes. Sample labels as in Tables 1 and 2.**

|  | HE | HH | LPA | NEA | EAM | AND | AS | OCE | IN | AFR | IB | **IE** | KHO | NE | UC | EUP | EUR |
| --- | --- | --- | --- | --- | --- | --- | --- | --- | --- | --- | --- | --- | --- | --- | --- | --- | --- |
| HE |  | 63,85 | 58,89 | 67,60 | 95,28 | 169,34 | 143,62 | 129,49 | 126,41 | 156,97 | 137,51 | **103,93** | 131,24 | 149,91 | 94,06 | 127,11 | 130,47 |
| HH | 48,86 |  | 28,42 | 19,43 | 62,60 | 123,81 | 127,18 | 86,24 | 119,50 | 118,58 | 113,42 | **135,86** | 104,91 | 109,33 | 69,51 | 100,69 | 89,26 |
| LPA | 44,93 | 20,01 |  | 25,25 | 23,64 | 69,01 | 67,10 | 42,29 | 65,64 | 60,79 | 57,71 | **113,52** | 42,41 | 61,22 | 30,36 | 42,22 | 45,91 |
| NEA | 54,64 | 13,04 | 19,27 |  | 57,29 | 104,84 | 103,92 | 79,15 | 99,76 | 104,52 | 99,07 | **100,16** | 96,53 | 87,89 | 56,45 | 84,21 | 76,69 |
| EAM | 78,44 | 51,43 | 15,51 | 48,68 |  | 20,51 | 25,11 | 11,42 | 26,64 | 22,14 | 17,66 | **101,56** | 16,08 | 24,94 | 11,69 | 12,42 | 17,90 |
| AND | 151,38 | 112,27 | 61,43 | 96,66 | 15,03 |  | 8,08 | 11,45 | 17,79 | 8,49 | 6,09 | **144,27** | 18,64 | 9,03 | 27,06 | 9,55 | 10,00 |
| AS | 127,32 | 115,59 | 59,79 | 95,95 | 19,50 | 6,69 |  | 15,15 | 7,39 | 13,25 | 4,16 | **133,36** | 19,59 | 5,58 | 27,75 | 8,78 | 8,95 |
| OCE | 113,82 | 76,86 | 36,24 | 72,43 | 6,42 | 9,67 | 13,30 |  | 19,53 | 10,62 | 8,70 | **131,69** | 14,15 | 16,00 | 17,18 | 6,45 | 11,90 |
| IN | 110,43 | 107,67 | 57,72 | 91,34 | 20,26 | 15,14 | 5,50 | 16,73 |  | 16,48 | 9,06 | **128,61** | 23,63 | 17,08 | 24,53 | 10,18 | 14,45 |
| AFR | 139,72 | 107,33 | 53,67 | 96,34 | 16,54 | 6,90 | 11,53 | 8,87 | 13,89 |  | 9,02 | **136,14** | 9,44 | 13,97 | 26,71 | 5,03 | 11,50 |
| IB | 120,95 | 102,02 | 50,33 | 90,76 | 11,88 | 4,20 | 2,53 | 6,61 | 6,46 | 6,94 |  | **134,10** | 15,91 | 10,69 | 16,97 | 4,88 | 9,72 |
| **IE** | **75,34** | **109,15** | **88,86** | **77,79** | **76,86** | **120,28** | **110,16** | **108,39** | **105,00** | **112,61** | **110,24** |  | **132,84** | **127,79** | **101,73** | **121,07** | **134,07** |
| KHO | 115,79 | 94,75 | 36,67 | 89,11 | 11,13 | 16,76 | 17,80 | 12,49 | 20,92 | 8,07 | 13,72 | **109,80** |  | 18,29 | 27,58 | 7,02 | 11,51 |
| NE | 132,88 | 98,42 | 53,88 | 80,49 | 18,97 | 7,22 | 4,12 | 13,73 | 14,26 | 11,85 | 8,31 | **104,56** | 16,21 |  | 30,89 | 11,60 | 3,90 |
| UC | 73,55 | 54,19 | 18,08 | 44,15 | -0,25 | 17,44 | 18,24 | 8,10 | 14,53 | 17,09 | 7,48 | **73,27** | 18,20 | 20,83 |  | 13,83 | 25,74 |
| EUP | 111,41 | 90,29 | 36,00 | 77,02 | 7,19 | 7,71 | 7,13 | 4,74 | 7,77 | 3,43 | 2,84 | **98,22** | 5,60 | 9,42 | 4,77 |  | 8,00 |
| EUR | 114,32 | 79,28 | 39,22 | 69,70 | 12,10 | 7,87 | 7,04 | 9,63 | 11,53 | 9,27 | 7,15 | **110,21** | 9,58 | 1,93 | 15,73 | 5,78 |  |
